# Activity of Anthracenediones and Flavoring Phenols in Hydromethanolic Extracts of *Rubia tinctorum* against Grapevine Phytopathogenic Fungi

**DOI:** 10.3390/plants10081527

**Published:** 2021-07-26

**Authors:** Natalia Langa-Lomba, Eva Sánchez-Hernández, Laura Buzón-Durán, Vicente González-García, José Casanova-Gascón, Jesús Martín-Gil, Pablo Martín-Ramos

**Affiliations:** 1 Instituto Universitario de Investigación en Ciencias Ambientales de Aragón (IUCA), EPS, Universidad de Zaragoza, Carretera de Cuarte, s/n, 22071 Huesca, Spain; natalialangalomba@gmail.com (N.L.-L.); jcasan@unizar.es (J.C.-G.); 2 Agrifood Research and Technology Centre of Aragón, Plant Protection Unit, Instituto Agroalimentario de Aragón—IA2 (CITA-Universidad de Zaragoza), Avda. Montañana 930, 50059 Zaragoza, Spain; vgonzalezg@aragon.es; 3 Department of Agricultural and Forestry Engineering, ETSIIAA, Universidad de Valladolid, 34004 Palencia, Spain; eva.sanchez.hernandez@uva.es (E.S.-H.); laura.buzon@uva.es (L.B.-D.); mgil@iaf.uva.es (J.M.-G.)

**Keywords:** antifungal, *Botryosphaeriaceae*, chitosan, GTDs, madder, stevioside, *Vitis vinifera*

## Abstract

In this work, the chemical composition of *Rubia tinctorum* root hydromethanolic extract was analyzed by GC–MS, and over 50 constituents were identified. The main phytochemicals were alizarin-related anthraquinones and flavoring phenol compounds. The antifungal activity of this extract, alone and in combination with chitosan oligomers (COS) or with stevioside, was evaluated against the pathogenic taxa *Diplodia seriata, Dothiorella viticola* and *Neofusicoccum parvum*, responsible for the so-called Botryosphaeria dieback of grapevine. In vitro mycelial growth inhibition tests showed remarkable activity for the pure extract, with EC_50_ and EC_90_ values as low as 66 and 88 μg·mL^−1^, respectively. Nonetheless, enhanced activity was attained upon the formation of conjugate complexes with COS or with stevioside, with synergy factors of up to 5.4 and 3.3, respectively, resulting in EC_50_ and EC_90_ values as low as 22 and 56 μg·mL^−1^, respectively. The conjugate with the best performance (COS-*R. tinctorum* extract) was then assayed ex situ on autoclaved grapevine wood against *D. seriata*, confirming its antifungal behavior on this plant material. Finally, the same conjugate was evaluated in greenhouse assays on grafted grapevine plants artificially inoculated with the three aforementioned fungal species, resulting in a significant reduction in the infection rate in all cases. This natural antifungal compound represents a promising alternative for developing sustainable control methods against grapevine trunk diseases.

## 1. Introduction

The joint presence of compounds of quinone and phenol categories in plant extracts and, specifically, the differential content of anthracenediones and 4-*tert*-butyl-2-phenyl-phenol, which might be responsible for the chromatic aberration of teak (difference between heartwood and sapwood), has been the object of attention in the bibliography [1].

**Figure 1:**
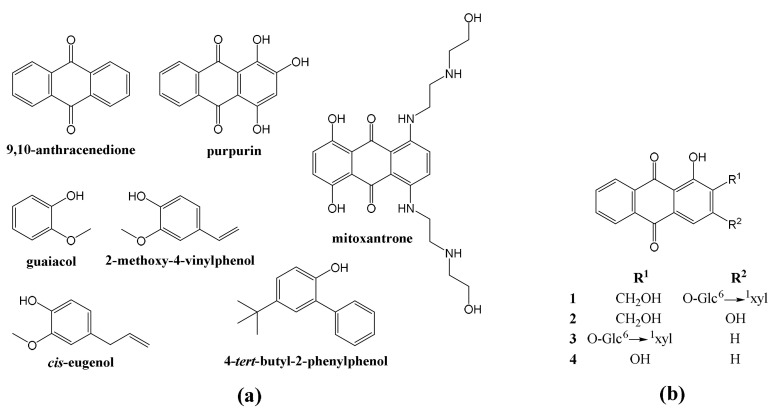
(**a**) Structures of different anthracenediones and phenols; (**b**) structures of alizarin-3-*O*-*β*-primeveroside, 3; lucidin-3-*O-β*-primeveroside, 1; and their aglycons (alizarin, 4; lucidin, 2). Glc, D-glucose; xyl, D-xylose.

Anthracenediones are a class of molecules based on the 9,10-anthracenedione parent (Figure 1a), which–among others–include purpurin (Figure 1a) and those synthesized by the American Cyanamid Laboratories in the late 1970s [2]. Although mitoxantrone, which has a dihydroxyanthraquinone central chromophore with two symmetrical aminoalkyl side chains (Figure 1a), is considered the biologically most active anthracenedione [3], other anthracenediones have also been reported to have antimicrobial activities: for instance, anthraquinone aglycones have been found to have a remarkable in vitro activity against clinical strains of dermatophytes [4]; anthraquinone derivatives exhibit antifungal activity against *Candida albicans* (C.P. Robin) Berkhout, *Cryptococcus* *neoformans* (San Felice) Vuill., *Trichophyton mentagrophytes* (C.P. Robin) R. Blanch., *Aspergillus fumigatus* Fresen. and *Sporothrix schenckii* Hektoen and C.F. Perkins [5,6]; purpurin possesses remarkable antifungal activity against *Candida* spp. [7]; and alizarin or 1,2-dihydroxyanthraquinone (Figure 1b) show antifungal behavior against *Aspergillus niger* Tieghem and *A. ochraceus* K. Wilhelm [8].

Flavoring phenols is a category that includes small free phenolic compounds (Figure 1a), such as 2-methoxy-phenol (or guaiacol), 2-methoxy-4-vinylphenol (or 4-vinyl-guaiacol), *cis*-2-methoxy -4-(1-propenyl)-phenol (or *cis*-eugenol) and 4-*tert*-butyl-2-phenyl-phenol, which participate in the aroma of wine. Guaiacol and eugenol are characterized by spice, clove, and smoke notes (guaiacol provides a roasted aroma and eugenol confers a clove aroma); and 4-vinyl-guaiacol has an odor reminiscent of carnation (*Dianthus* flowers). 4-((1E)-3-hydroxy-1-propenyl)-2-methoxyphenol (or coniferyl alcohol) is a precursor of grape and wine volatiles [9]. All of them are present in oak, but 4-*tert*-butyl-2-phenyl-phenol has been referred as a constituent of *Rubia cordifolia* L. essential oil [10,11]. Regarding their antifungal activities, a strong antifungal activity against *Botrytis cinerea* Pers. has been referred for eugenol [12], and guaiacol has been found to be effective against sap-staining fungi (*Ophiostoma* spp.) [13]. 

In this paper, the possibility of a joint presence of both anthracenediones and flavoring phenols in *Rubia tinctorum* L. (*Rubiaceae*) has been explored, given that the presence of 9,10-anthraquinones and other biologically active compounds has been reported for other members of the genus *Rubia*, mainly for *R. cordifolia*, as summarized in the review paper by Singh, et al. [14]. 

*R. tinctorum* is widely distributed in southern and southeastern Europe, in the Mediterranean area, and in central Asia. Its reddish roots contain hydroxyanthraquinones, such as alizarin (used for the dyeing of textiles [15] and in the treatment of kidney and bladder stones), purpurin (1,2,4-trihydroxyanthraquinone), and lucidin (Figure 1b.4) [16,17]; and flavoring phenols such as 4-vinyl-guaiacol [18]. 

The interest in the joint presence of anthracenediones and phenols (as 2-methoxyphenols and 4-*tert*-butyl-2-phenyl-phenol) lies in the possibility of synergies that enhance their microbiological activity. In particular, this work focuses on their potential application for the control of grapevine trunk diseases (GTDs), currently considered one of the most relevant challenges in Viticulture, as these pathologies cause significant economic losses in grape growing areas all over the world. Under this generic concept, a series of mycoses are grouped, which affect the wood of grapevine throughout its entire life cycle [19,20]. Among them, those that affect young plants coming from the nursery and in the first years after planting are especially important from the economic point of view, being responsible for numerous losses derived from the removal and replacement of plants in hundreds of thousands of hectares around the world [21]. Some of these include the so-called "Black Foot" disease, caused by different species belonging to soil-borne genera like *Ilyonectria*, *Campylocarpon*, *Cylindrocladiella*, *Dactylonectria*, etc.; the etiological agents responsible for Petri disease (mainly species of the genus *Phaeoacremonium*, and *Phaeomoniella chlamydospora* (W. Gams, Crous, M.J. Wingf. and Mugnai) Crous and W. Gams) that for many authors would be part of the first stages of the complex esca syndrome; or some species of the ascomycete family *Botryosphaeriaceae*, especially certain aggressive taxa in the early years of the plant such as *Neofusicoccum parvum* (included in the present study). In addition to these pathologies, other complex syndromes have been described, such as the aforementioned esca (attributable to certain species of lignicolous basidiomycetes), Eutypiosis (caused in Europe by *Eutypa lata* (Pers.) Tul. and C. Tul.), or the so-called Botryosphaeria decay of grapevine plants (also known as "Black Dead Arm" disease) caused by various genera and species of this family such as the aforementioned *N. parvum*, *Diplodia* spp., *Dothiorella* spp., *Lasiodiplodia* spp. or *Botryosphaeria* spp. 

Given that the prohibition of active ingredients such as sodium arsenite and benzimidazoles, which were used to control GTDs, has worsened the impact of these diseases, they have become the subject of intense research efforts. Unfortunately, due to the breadth and complexity of the problem, no single effective control measure against these mycoses has been developed to date. Current strategies and future prospects for the management of GTDs are thoroughly discussed in the review papers by Fontaine, et al. [22], Bertsch, et al. [20], Mondello, et al. [23] and Gramaje, et al. [24], but the use of active ingredients of natural origin, instead of conventional chemicals, poses an especially interesting approach, aligned with the criteria of European legislation currently in force (Article 14 in European Directive 2009/128/EC).

Taking into consideration that many phytochemicals have solubility and bioavailability problems, in this work the bioactivity of the hydromethanolic extracts of *R. tinctorum* against GTDs has also been assayed after the formation of conjugate complexes, either with chitosan oligomers (COS) or with stevioside [a terpene glycoside obtained from *Stevia rebaudiana* (Bertoni) Bertoni extract], which also have antifungal properties and which may lead to a synergistic fungicide behaviour [25,26].

## 2. Material and Methods

### 2.1. Plant Material and Chemicals

The specimens of *Rubia tinctorum* under study were collected on the banks of the Carrión river as it passes through the town of Palencia (Spain). The roots were shade-dried and pulverized to fine powder in a mechanical grinder. Samples from different specimens (*n* = 25) were thoroughly mixed to obtain composite samples.

Chitosan (CAS 9012-76-4; high MW: 310,000–375,000 Da) was supplied by Hangzhou Simit Chem. & Tech. Co. (Hangzhou, China). Neutrase^TM^ 0.8 L enzyme was supplied by Novozymes A/S (Bagsværd, Denmark). Stevioside (CAS 57817-89-7, 99%) was purchased from Wako Chemicals GmbH (Neuss, Germany). Quantities of 4-*tert*-butyl-2-phenylphenol (CAS 98-27-1, 97%), 1,2-dihydroxyanthraquinone (CAS 72-48-0, 97%), sodium alginate (CAS 9005-38-3), calcium carbonate (CAS 471-34-1, ≥99.0%) and methanol (CAS 67-56-1, UHPLC, suitable for mass spectrometry) were acquired from Sigma-Aldrich Química (Madrid, Spain). Agar (CAS 9002-18-0) and PDA (potato dextrose agar) were supplied by Becton Dickinson (Bergen County, NJ, USA). 

### 2.2. Preparation and Physicochemical Characterization of the of R. tinctorum Extracts

*Rubia tinctorum* samples were mixed (1:20, *w*/*v*) with a methanol/water solution (1:1 *v*/*v*) and heated in a water bath at 50 °C for 30 min, followed by sonication for 5 min in pulse mode with a 1 min stop for each 2.5 min, using a 1000 W probe-type ultrasonicator operated at 20 kHz (model UIP1000hdT, Hielscher Ultrasonics, Teltow, Germany). The solution was then centrifuged at 9000 rpm for 15 min and the supernatant was filtered through Whatman No. 1 paper. Aliquots were lyophilized for the vibrational spectroscopy analysis.

The infrared vibrational spectra of both dried and ground roots and the lyophilized extract were registered using a Thermo Scientific (Waltham, MA, USA) Nicolet iS50 Fourier-transform infrared spectrometer, equipped with an in-built diamond attenuated total reflection (ATR) system. The spectra were collected with a 1 cm^-1^ spectral resolution over the 400–4000 cm^−1^ range, taking the interferograms that resulted from co-adding 64 scans. The spectra were then corrected using the advanced ATR correction algorithm [27] available in OMNIC^TM^ software suite.

The hydroalcoholic plant extract was studied by gas chromatography–mass spectrometry (GC–MS) at the Research Support Services (STI) at Universidad de Alicante (Alicante, Spain), using a gas chromatograph model 7890A coupled to a quadrupole mass spectrometer model 5975C (both from Agilent Technologies). The chromatographic conditions were: 3 injections/vial, injection volume = 1 µL; injector temperature = 280 °C, in splitless mode; initial oven temperature = 60 °C, 2 min, followed by ramp-up of 10 °C/min to a final temperature of 300 °C, 15 min. The chromatographic column used for the separation of the compounds was an Agilent Technologies HP-5MS UI of 30 m length, 0.250 mm diameter and 0.25 µm film. The mass spectrometer conditions were: temperature of the electron impact source of the mass spectrometer = 230 °C and of the quadrupole = 150 °C; ionization energy = 70 eV. Test mixture 2 for apolar capillary columns according to Grob (Supelco 86501) and PFTBA tuning standards were used for equipment calibration. NIST11 library and the monograph by Adams [28] were used for compound identification.

### 2.3. Preparation of Chitosan Oligomers and Bioactive Formulations

Chitosan oligomers (COS) were prepared according to the procedure reported by Santos-Moriano, et al. [29], with the modifications indicated in [30], obtaining oligomers with a molecular weight <2000 Da. 

The COS-*R. tinctorum* and stevioside–*R. tinctorum* conjugate complexes were obtained by mixing the respective solutions in a 1:1 (*v*/*v*) ratio. The mixtures were then sonicated for 15 min in five 3-minute periods (so that the temperature did not exceed 60 °C) using a probe-type ultrasonicator.

For the assays carried out on autoclaved wood, the conjugate complex was dispersed in an agar matrix (15 g/L in Milli-Q water), using a procedure analogous to the one described below for the in vitro tests.

For the in vivo assays, the bioactive product was dispersed in a calcium alginate matrix. Hydrogel beads were prepared as follows: the control product was added to a 3% sodium alginate solution in a 2:8 ratio (20 mL compound/80 mL sodium alginate). Subsequently, this solution was dispensed drop by drop onto a 3% calcium carbonate solution to polymerize (30 min curing), obtaining beads with diameters in the 4–6 mm range.

### 2.4. Fungal Isolates

The three fungal isolates used (Table 1) were supplied as lyophilized vials (later reconstituted and refreshed as PDA subcultures) by the Agricultural Technological Institute of Castilla and Leon (ITACYL, Valladolid, Spain) [31].

### 2.5. Antifungal Activity Assessment

#### 2.5.1. In vitro Tests of Mycelial Growth Inhibition

The antifungal activity of the different treatments was determined using the agar dilution method according to EUCAST standard antifungal susceptibility testing procedures [32], by incorporating aliquots of stock solutions onto the PDA medium to obtain concentrations in the 15.62–1500 μg·mL^−1^ range. Mycelial plugs (∅ = 5 mm) from the margin of 1-week-old PDA cultures of *D. seriata*, *D. viticola* or *N. parvum* were transferred to plates incorporating the above-mentioned concentrations for each treatment (3 plates per treatment/concentration, with 2 replicates each). Plates were then incubated at 25 °C in the dark for a week. PDA medium without any amendment was used as control. Mycelial growth inhibition was estimated according to the formula: ((*d_c_* − *d_t_*)/*d_c_)* × 100, where *d_c_* and *d_t_* represent the average diameters of the fungal colony of the control and of the treated fungal colony, respectively. Effective concentrations (EC_50_ and EC_90_) were estimated using PROBIT analysis in IBM SPSS Statistics v.25 (IBM; Armonk, NY, USA) software. The level of interaction (i.e., synergy factors) was determined according to Wadley’s method [33].

#### 2.5.2. Assays on Autoclaved Grape Wood

The formulation (COS-*R. tinctorum* conjugate) that showed the best performance in the in vitro assays was then tested on autoclaved grapevine wood to assess its behaviour on plant material against the least sensitive fungus in the previous plate tests. One-year-old dormant canes (*Vitis vinifera* L. cv. ‘Tempranillo’) were cut into 16 cm (length) and 0.8–1 cm (diameter) segments and autoclaved twice at 121 °C (20 min) to eliminate any microbial contamination. Inoculation was performed by first making two approximately 3 mm deep slits with a scalpel (without reaching the medullary tissue) per shoot, 8–10 cm apart and located in the internodes. A 3 mm diameter plug of PDA agar coming from the margin of a 10-day colony of the pathogen (*D. seriata*) was placed in each slit, flanked by 2 plugs (∅ = 3 mm) of bacteriological agar that contained the tested conjugate complex. After this, the wounds were covered with autoclaved cotton moistened with sterile bi-distilled water and sealed with Parafilm^TM^ tape. Inoculated shoots were placed in transparent culture boxes on a bed of sterile filter paper, periodically moistened (with sterile double distilled water), and incubated for 21 days in a climatic chamber at 26 °C, with 70% RH and a 12/12 h photoperiod. A total of 5 boxes with 3 replicates/box each were arranged, together with a positive control inoculated only with *D. seriata* (1 box with 3 replicates) and a negative control without pathogen, inoculated only with the conjugate (also 1 box with 3 replicates).

After the incubation period, segments were recovered from the boxes, and each of them was divided into two halves of approximately 8 cm, before longitudinal cuts were made in each half. Finally, the length of the vascular necroses produced was measured longitudinally on upper and lower directions from the inoculation point for both halves, and compared with those of controls.

#### 2.5.3. Greenhouse Bioassays on Grafted Plants

Bioassays with COS (chosen as a reference) and COS-*R. tinctorum* conjugate complexes were performed in living plants in order to scale the protective capabilities of these compounds against the three selected *Botryosphaeriaceae* species in young grapevine plants. As summarized in Table S1, plant material consisted of 30 plants of ‘Tempranillo’ (CL. 32 clone) (2 year old) cultivar and 30 plants of ‘Garnacha’ (VCR3 clone) (1 year old) cultivar, grafted on 775P and 110R rootstocks, respectively. Each plant was cultured on a 3.5 L plastic pot containing a mixed substrate of moss peat and sterilized natural soil (75:25), incorporating slow release fertilizer when needed along the culture cycle. Plants were maintained in the greenhouse with drip irrigation and anti-weed ground cover from June to December 2020 (6 months). One week after placing them in pots, young, grafted plants were artificially inoculated with the pathogens and the COS-*R. tinctorum* treatment. Five repetitions (plants) were arranged for each pathogen*cultivar combination, together with 4 positive controls/(pathogen*cultivar) plus 3 negative controls (incorporating only the bioactive product) for each cultivar. Inoculations of both pathogens and the control product were carried out directly on the trunk of the living plants at two sites per stem (separated >5 cm) below the grafting point and not reaching the root crown. For the different fungi, agar plugs from the margin of 5 day old fresh PDA cultures of each species were used as fungal inoculum. In the aforementioned two inoculation points of each grapevine plant, slits of approx. 15 mm in diameter and 5 mm deep were made with a scalpel. Subsequently, 5 mm diameter agar plugs were placed directly into contact with vascular tissue in the stem; simultaneously, calcium alginate hydrogel beads containing the bioactive product were placed at both sides of the agar plug; and the whole set was covered with cotton soaked in sterile bi-distilled water and sealed with Parafilm^TM^ tape. During the culture period, application of copper (cuprous oxide 75%, Cobre Nordox^TM^ 75 WG) to control downy mildew outbreaks was performed in mid-July, accompanied with a first sprouting (followed by periodic sprouting). Plants were visually examined weekly for the presence of foliar symptoms. After six months in the greenhouse, the plants were removed, two sections of the inoculated stems between the grafting point and the root crown were prepared and sectioned longitudinally. The length of the vascular necroses was scored longitudinally on upper and lower directions from the inoculation point for both halves of the longitudinal cut, and the average measures of these were statistically analysed and compared depending on the type of pathogen. All the data were compared with positive and negative controls. Finally, grapevine plants removed and measured at the end of the assay were also processed to re-isolate the different pathogenic taxa previously inoculated. Thus, 5 mm long wood chips exhibiting vascular necroses (1–2 cm around the wounds) were washed, their surface sterilized, then placed in PDA plates amended with streptomycin sulphate (to avoid bacterial contamination) and incubated in a culture chamber at 26 °C in the dark for 2–3 days.

### 2.6. Statistical Analyses

The results of the in vitro inhibition of mycelial growth were statistically analyzed using one-way analysis of variance (ANOVA), followed by post hoc comparison of means through Tukey’s test at *p* < 0.05 (provided that the homogeneity and homoscedasticity requirements were satisfied, according to the Shapiro–Wilk and Levene tests). In the case of autoclaved grapevine wood and greenhouse assay results, since the normality and homoscedasticity requirements were not met, the Kruskal–Wallis non-parametric test was used instead, with the Conover–Iman test for post hoc multiple pairwise comparisons. R statistical software was used for all the statistical analyses [34].

## 3. Results

### 3.1. Vibrational Characterization

The assignment of the main absorption bands in the infrared spectra of the *R. tinctorum* root powder and root extracts is shown in Table 2. The most prominent band, attributed to the benzene ring in aromatic compounds, occurs at ca. 1500 cm^−1^. The bands at 1592 cm^−1^ and 1676 cm^−1^ can be assigned to the in-phase C=O and symmetrical C=C vibrations from anthraquinone.

### 3.2. Gas Chromatography–Mass Spectrometry Analysis of the Extract

In *R. tinctorum* root hydromethanolic extracts, the main analyzed components (Table 3) were: the anthraquinone family (19.4%) consisting of 2-methyl-9,10-anthracenedione (or *β*-methylanthraquinone) (15.5%), 1,2-dihydroxyanthraquinone (or alizarin), 1,8-dihydroxy-3-methylanthraquinone, 1-hydroxy-9,10-anthracenedione (or *α*-hydroxyanthraquinone) and 1-hydroxy-4-methylanthraquinone; cyclopentenones (2.3%), such as 2-hydroxy-2-cyclopenten-1-one and 4-cyclopentene-1,3-dione; and the phenol category (7.5%), constituted by *cis*-2-methoxy-4-(1-propenyl)-phenol (or *cis*-eugenol), 2-methoxy-phenol (or guaiacol), 2-methoxy-4-vinylphenol (or 4-vinyl-guaiacol), 4-*tert*-butyl-2-phenylphenol, and coniferyl alcohol. Other phytochemicals of interest were 4-methoxy-4',5'-methylenedioxybiphenyl-2-carboxylic acid (8.6%), 1,4-diacetyl-3-acetoxymethyl-2,5-methylene-l-rhamnitol (8.3%) and guanosine (5.8%).

It is worth noting that the flavoring phenols found in the hydroalcoholic extracts from *R. tinctorum* (guaiacol, 4-vinyl-guaiacol and *cis*-eugenol) were the same present in oak, which are used to confer aroma to wine. 

### 3.3. Antifungal Activity

#### 3.3.1. In vitro Tests of Mycelial Growth Inhibition

**Figure 2:**
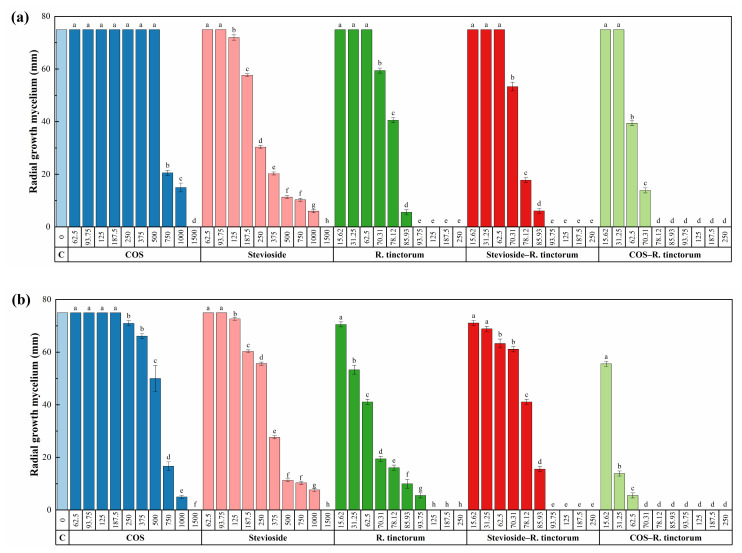

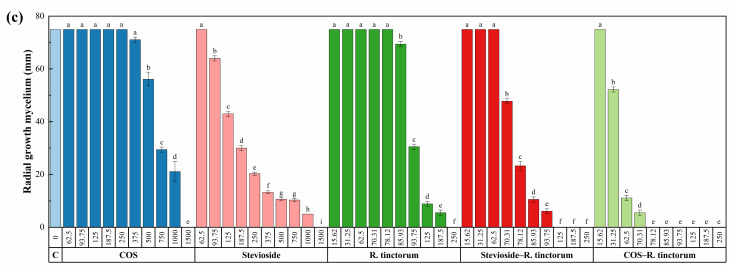
Colony growth measures of (**a**) *D. seriata*, (**b**) *D. viticola* and (**c**) *N. parvum* strains when cultured in PDA plates containing the various control products (viz. chitosan oligomers (COS), stevioside, *R. tinctorum* hydromethanolic extract, stevioside−*R. tinctorum* and COS-*R. tinctorum* conjugate complexes) at concentrations in the 62.5−1500 and 15.62−250 μg·mL^−1^ range ordered according to the least and the most active products, respectively. The same letters above concentrations indicate that they are not significantly different at *p* < 0.05. Error bars represent standard deviations.

The results of the mycelial growth inhibition tests for the hydromethanolic *R. tinctorum* root extract, alone or forming a conjugate complex with COS or stevioside, are presented in Figure 2 and Figures S2–S4. The antifungal activity of the extract was found to be much higher than those of COS and stevioside alone, reaching full inhibition at concentrations in the 93.8–250 μg·mL^−1^ range, depending on the pathogen (vs. 1500 μg·mL^−1^ for COS and stevioside). Upon conjugation with stevioside, some improvement in the germicide effect could be observed: for instance, the inhibition of *D. seriata* was higher at the 78.1 μg·mL^−1^ concentration (76.3% vs. 45.9%), and the full inhibition of *D. viticola* and *N. parvum* was attained at a lower concentration (93.8 vs. 125 μg·mL^−1^, and 125 vs. 250 μg·mL^−1^, respectively). Nonetheless, the best results were obtained for the COS−*R. tinctorum* extracts, for which full inhibition was recorded at the lowest concentrations (in the 70.3–78.1 μg·mL^−1^ range).

In order to provide a tentative explanation for the strong antifungal activity observed in the extracts, three of the presumably bioactive constituents were also assayed (an anthracenedione, a phenol and a purine nucleoside) separately. The results, presented in Figures S5–S8, showed that 4-*tert*-butyl-2-phenylphenol was the most active (full inhibition of the three fungi was attained at concentrations in the 78.1–93.8 μg·mL^−1^ range), but 1,2,4-trihydroxyanthraquinone and guanosine were also effective (full inhibition was reached at concentrations in the 187.5–500 and 250–375 μg·mL^−1^ ranges, respectively). Such values are comparable to those found for the whole *R. tinctorum* extract, suggesting that the activity cannot be ascribed to a single constituent, but rather to the combination of several of them.

To quantify the synergistic behavior observed for the conjugate complexes, effective concentrations were estimated (Table 4) and synergy factors (SF) were then calculated according to Wadley’s method (Table 5). As expected, the synergism between COS and *R. tinctorum* extract was noticeably higher than the one observed between stevioside and *R. tinctorum* extract, with SF values in the 2.23–5.35 and 1.36–3.29 range, respectively.

#### 3.3.2. Assays on Autoclaved Grapevine Wood

The results from the ex situ experiment conducted on autoclaved grapevine canes for the most promising treatment (COS-*R. tinctorum* conjugate complex) and the least sensitive fungus (*D. seriata*), presented in Table 6, showed that the application of the bioactive product led to statistically significant differences in terms of vascular necroses vs. the positive control. Nonetheless, it did not lead to full inhibition, given that there were statistically significant differences in the length of vascular lesions compared with the negative control (shoots inoculated only with the bioactive compound). This could be tentatively attributed to the chosen dispersion medium (agar), which was replaced with calcium alginate in subsequent in vivo experiments.

#### 3.3.3. Greenhouse Bioassays on Grafted Plants

When the best treatment (COS-*R. tinctorum* conjugate complex) was further assayed in vivo, significant differences were found against the positive controls in all cases (Table 7), confirming its antifungal behavior on the plant material. Nonetheless, complete inhibition was not reached against any of the three pathogens for the assayed dose (100 μg·mL^−1^) comparing with non-infected controls, suggesting that a higher concentration than the EC_90_ values found in the in vitro tests (and/or a different dispersion medium) should be assayed when the treatment is used in future field trials.

## 4. Discussion

### 4.1. On the Constituents of R. tinctorum Extracts

The composition here reported was different from that found by Derksen and Van Beek [35] (using LC–DAD and HPLC–MS(/MS) with ESI or APCI), where lucidin primeveroside and ruberythric acid were the major anthraquinone components in an ethanolic-water extract, and from the one reported by Jalill [18] for a methanolic extract, which was rich in 9,12-octadecadienoic acid (29.75%), 9-octadecenoic acid hexadecyl ester (26.1%) and 2-ethyl-2-(hydroxymethyl)-1,3-propanediol, (10.1%), but poor in anthracenediones (4.0%) and 2-methoxy-4-vinylphenol (0.5%).

Significant differences in composition were also observed in comparison with the *Rubia cordifolia* essential oil characterized by GC–MS, in which mollugin (rubimaillin or methyl 6-hydroxy-2,2-dimethylbenzo[h]chromene-5-carboxylate) was found to be the major component, followed by 3-methyl-2-cyclopenten-1-one, eugenol, anethole and 4-*tert*-butyl-2-phenylphenol [10,11]. 

Although the geographical location, time of year and age of the plant are known to influence the composition [15], the observed differences should be mainly ascribed to differences in both the extractive chemicals and in the extraction process (nature of the alcoholic solvent, alcohol:water ratio and mechanical enhancers such as sonication [36,37]), and to the characterization technique, provided that previous studies on *R. tinctorum* extracts [36,38,39,40,41] were conducted by HPLC and LC–HRMS (instead of GC–MS) and generally focused only on anthraquinones, anthraquinone glycosides and aglycones. 

### 4.2. On the Combined Effect of Anthraquinones and Phenols

It is known that increasing the activity of a parent molecule can be pursued either by testing multiple substituent changes on the base core (the impact of the number, nature, and location of substituents on the anthraquinone moiety on its inhibitory potency against pathogenic fungi has been studied in [42]), or by testing the effect of coexistence with other molecules with which synergistic behavior may occur. In general, anthraquinone per se is a relatively inert compound, but in the presence of glucose, anthrahydroquinone units (formed by reduction of anthraquinone) reduce the quinone–methide units (issued by dehydration of phenolic *β*-*O*-4 lignin) mainly by electron transfer leading to guaiacol [43]. Thus, the presence of 4-vinyl-guaiacol, *cis*-eugenol, coniferyl alcohol or 4-*tert*-butyl-2-phenylphenol phytochemicals in the *R. tinctorum* hydromethanolic extract should be referred to the same origin. As regards a subsequent interaction of these phenols with anthraquinones, it cannot be excluded: Maurino et al. [44] have demonstrated that quinonoid compounds excited by sunlight react with phenols, transforming them into tetrasubstituted dihydroxybiphenyls and phenoxyphenols. Nevertheless, in the absence of induced sunlight, no reaction between anthraquinones and methoxy- and phenyl-phenols has been described in the literature (to the best of the authors’ knowledge), so at this point it is not possible to establish whether the activity of *R. tinctorum* extracts may be referred to an additive effect of both families of components or to a synergistic one. 

### 4.3. Comparison with Efficacies Reported in the Literature

An overview of the antimicrobial activities reported for *R. tinctorum* in the literature is presented in Table S2. Concerning its antifungal behavior, full inhibition of *Aspergillus flavus* Link and *Fusarium oxysporum* Schltdl. at a concentration of 100 μg·mL^−1^ has been reported by Kalyoncu, et al. [45], and inhibition percentages in the 18–43% range were reported against *Trichoderma viride* Pers., *Doratomyces stemonitis* (Pers.) Nees, *Aspergillus niger*, *Penicillium verrucosum* Dierckx, *Alternaria alternate* (Fr.) Keissl., *Aureobasidium pullulans* (de Bary) G. Arnaud and *Mucor mucedo* L. by Manojlovic et al. [46], although the assayed concentration was not reported. Activity against other fungi (e.g., *Penicillium expansum* Link, *Geotrichum candidum* Link, *Fusarium solani* (Mart.) Sacc., *Postia placenta* (Fr.) M.J. Larsen and Lombard, *Trametes versicolor* (L.) Lloyd) has also been reported, albeit not in a quantitative manner [47,48].

The contribution of anthraquinones to antifungal activity is well-established, given that anthracenediones from other plants have proven to be effective against a wide variety of phytopathogenic fungi. For instance, anthraquinones isolated from *Cassia tora* L., *Coccoloba mollis* Casar., *Rheum palmatum* L., *Morinda lucida* Benth. or *Aegle marmelos* (L.) Corrêa, to name a few, showed antifungal behavior against phytopathogenic fungi such as *Botrytis cinerea*, *Blumeria graminis* (DC.) Speer, *Phytophthora infestans* (Mont.) de Bary, *Puccinia recondita* Roberge ex Desm., *Pyricularia grisea* Sacc., *Rhizoctonia solani* J.G. Kühn, *Botryospheria ribis* Grossenbacher and Duggar, *B. rhodina* (Berk. and M.A. Curtis) Arx, *Lasiodiplodia theobromae* (Pat.) Griffon and Maubl., *Fusarium* sp., *Fusarium graminearum* Schwabe, *Mycosphaerella melonis* (Pass.) W.F. Chiu and J.C. Walker, *Fusarium oxysporum* f. sp. *vasinfectum* (G.F. Atk.) W.C. Snyder and H.N. Hansen, *Phyllosticta zeae* Stout, *Sclerotinia sclerotiorum* (Lib.) de Bary, *Cladosporium cucumerinum* Ellis and Arthur and *Aspergillus* spp. [49,50,51,52,53]. The underlying mechanism of action has been studied, for example, for purpurin against *Candida* spp., finding that it elevates intracellular ROS levels, depolarizes the mitochondrial membrane potential, downregulates of the expression of hypha-specific genes and the central morphogenetic regulator Ras1p and degrades DNA [54,55].

On the other hand, the antifungal activities of 2-methoxy- and 2-*tert*-butyl-substituted phenols against phytopathogens have been less studied, although a strong antifungal activity of 2-methoxy-4-(1-propenyl)-phenol against *Botrytis cinerea* was reported by Wang et al. [12]; against *B. rhodina, Rhizoctonia* sp. and *Alternaria* sp. by de Oliveira Pereira et al. [56]; and against *A. alternata* (Fr.) Keissl., *Sarocladium oryzae* (Sawada) W. Gams and D. Hawksw., *F. graminearum, F. equiseti* (Corda) Sacc. and *F. verticillioides* (Sacc.) Nirenberg by Pilar Santamarina et al. [57]. Likewise, 2-methoxy-phenol was effective against sap-staining fungi (*Ophiostoma* spp.), according to Velmurugan et al. [13]. Regarding their mechanism of action, it has been proposed that, for instance, eugenol acts on cell membrane by a mechanism that seems to involve the inhibition of ergosterol biosynthesis, and the lower ergosterol content interferes with the integrity and functionality of the cell membrane [56]. It has also been suggested that, taking into consideration that it induced the generation of H_2_O_2_ and increased free Ca^2+^ in the cytoplasm, its activity may also be referred to membrane binding and permeability alteration, leading to the destabilization and disruption of the plasma membrane [12].

### 4.4. On the Synergistic Behaviour of R. tinctorum Extracts with COS and Stevioside 

To date, it has been verified that chitosan acts as an elicitor on *R. tinctorum*, stimulating anthraquinone synthesis [58]; chitosan/poly (lactic acid) nanoparticles have been evaluated as a novel carrier for the delivery of anthraquinone [59]; and chitosan-based hydrogels have been studied for the adsorption of anthraquinone dyes [60]. Nonetheless, after a thorough bibliographical survey, no previous examples of the use of chitosan or stevioside for the formation of conjugate complexes with anthraquinones could be found. 

On the other hand, examples of synergistic behaviour have been reported, for instance, for chitosan combined with *Cinnamomum zeylanicum* Blume essential oils, rich in eugenol [61]. These authors hypothesized that eugenol alters the surface and structure of the fungal cell wall, and COS acts as a potentiator by reducing cell wall synthesis and facilitating death in an energy-dependent manner. In this regard, the accepted and potential mechanisms of action behind the antimicrobial properties of chitosan have been thoroughly discussed in the review paper by Ma et al. [62]. Those of stevioside have been discussed in [63], and are related to the uncoupling of mitochondrial oxidative phosphorylation and the permeabilization of the cell membrane.

Nonetheless, taking into consideration that the antifungal activity of both COS and stevioside alone was substantially lower than that of the *R. tinctorum* extract, and given that the use of most free anthraquinones in pharmaceutical industries is limited by their poor water solubility and low bioavailability [64], the observed strong synergistic behavior with COS and stevioside should probably be referred to a solubility and bioavailability enhancement through the formation of inclusion compounds or conjugate complexes (discussed, in the case of chitosan, in the recent review paper by Detsi et al. [65] and, for steviol glycosides, in the works by Nguyen et al. [66,67]). Examples of antifungal activity enhancement via the formation of conjugate complexes against GTDs have been previously reported in [25,26,68], albeit with worse EC_50_ and EC_90_ values than those reported in this work.

## 5. Conclusions

The GC–MS analysis of *R. tinctorum* hydroalcoholic extracts revealed that, apart from members of the anthraquinone family (19.4%), flavoring phenols similar to those found in oak (used to confer aroma to wine) and guanosine were also present. *R. tinctorum* extract, alone and forming conjugate complexes with COS and stevioside, along with three of its constituents, were assayed in vitro against three *Botryosphaeriaceae* taxa. *R. tinctorum* extract led to a strong mycelial growth inhibitory effect in all cases, with EC_90_ values as 88 μg·mL^−1^. Although 4-*tert*-butyl-2-phenylphenol was its most active constituent, 1,2,4-trihydroxyanthraquinone and guanosine were also effective, suggesting the activity cannot be ascribed to a single constituent, but rather to the combination of several of them. As regards the strong synergistic behavior observed upon conjugation with COS, which resulted in EC_90_ values in the 56–73 μg·mL^−1^ range, it may be ascribed to solubility and bioavailability enhancement, rather than to the antifungal activity of chitosan (which is much weaker than that of *R. tinctorum*). The treatment for which the best results were attained in plate tests (COS-*R. tinctorum* conjugate complex) was then tested ex situ on autoclaved grapevine twigs and in young, grafted plants in greenhouse assays. A significant reduction in the infection rate was found in all cases. Hence, this natural antifungal compound may deserve further examination in larger field trials, as it may be hold promise for the sustainable control of GTDs.

## Figures and Tables

**Table 1:** Fungal isolates used in the study.

Code	Isolate	Binomial Nomenclature	Geographical Origin	Host/Date
ITACYL_F098	Y-084-01-01a	*Diplodia seriata* De Not.	Spain (DO Toro)	Grapevine (‘Tempranillo’) 2004
ITACYL_F118	Y-103-08-01	*Dothiorella viticola* A.J.L.Phillips and J.Luque	Spain (Extremadura)	Grapevine 2004
ITACYL_F111	Y-091-03-01c	*Neofusicoccum parvum* (Pennycook and Samuels) Crous, Slippers and A.J.L.Phillips	Spain (Navarra, nursery)	Grapevine (‘Verdejo’) 2006

**Table 2:** Main bands in the infrared spectra of root and lyophilized *R. tinctorum* extract and of two of its main constituents.

*R. tinctorum*		Anthraquinone	4-tert-butyl-2-phenylphenol	Assignment
Root Powder	Extract			
3334	3335			Bonded O–H stretching (cellulose)
		2964		sp^3^ C–H
2920	2920	2925		=C–H groups of aromatic rings
		2856		aliphatic C–H asymmetrical stretching
		2724		β–OH, typical of α-hydroxy anthraquinone
1727	1733			C=O from esters
		1704		ester C=O
		1676		C=O in anthraquinones
1639	1620	1633		C=O in anthraquinones
1602	1605	1592	1585	phenyl ring (aromatic skeletal vibration) >C=C< in anthraquinones
1552			1545	carboxylate stretches/C=C aromatic
	1511		1480	methylene C–H bend
		1461	1470	methyl C–H asymmetrical
	1435		1430	=C–H in plane bending
1414	1416		1420	vinyl C–H in plane bending
1370	1406 1370	1377 1366	1385	C–C asymmetrical stretching phenolic hydroxyl groups
			1355	C–O stretching/methylene C–H bending
		1333 1329	1325	C–H in-plane deformation methylene C–H bending
1316	1316	1306		vinylidene C–H in plane bending
1255	1255	1287	1270	C–O stretching/C=C symmetric stretching
		1207	1215	C–O stretching/C–H in plane bending
		1171	1180	–C–O–C– stretching
	1142	1153	1135	
	1100	1099		
		1087	1080	
1020			1025	C–C stretching
	951	969		C–H out-of-plane bending

**Table 3:** Phytochemicals identified in *R. tinctorum* root hydromethanolic extract by GC–MS.

Peak	R_t_ (min)	Area (%)	Assignments
1	4.6369	1.99	4-pentenoic acid, ethyl ester
2	4.7440	0.26	l-gala-l-ido-octose
3	4.8414	0.52	4-cyclopentene-1,3-dione
4	5.0021	1.09	oxime-, methoxy-phenyl-
5	5.1968	0.46	1-(2-furanyl)-ethanone
6	5.2942	1.36	2,5-diethenyltetrahydro-2-methyl-furan
7	5.3770	1.82	2-hydroxy-2-cyclopenten-1-one
8	6.0781	0.70	2,4-dihydroxy-2,5-dimethyl-3(2H)-furan-3-one
9	6.3312	2.91	2-hydroxy-*γ*-butyrolactone
10	6.6331	2.47	glycerin
11	6.7694	1.93	1,2-cyclopentanedione, 3-methyl-
12	6.9690	1.19	2-acetamido-2-deoxy-α-D-glucopyranose
13	7.1345	0.76	butyronitrile, 4-ethoxy-3-hydroxy-
14	7.2952	1.55	2,5-dimethyl-4-hydroxy-3(2H)-furanone
15	7.4267	0.77	trimethyl(tetrahydrofuran-2-ylperoxy)silane
16	7.6798	1.01	2-methoxy-phenol (or guaiacol)
17	7.7869	2.20	L-alanine, methyl ester
18	8.2008	2.65	dimethyl dl-malate
19	8.3955	0.64	ethanamine, N-ethyl-N-nitroso-
20	8.5270	2.47	4H-pyran-4-one, 2,3-dihydro-3,5-dihydroxy-6-methyl-
21	9.1453	0.87	4H-pyran-4-one, 3,5-dihydroxy-2-methyl-
22	9.2865	0.97	catechol
23	9.4471	0.94	1,4:3,6-dianhydro-α-d-glucopyranose
24	9.6857	0.35	5-hydroxymethylfurfural
25	10.3965	0.31	2-acetoxy-5-hydroxyacetophenone
26	10.5718	0.50	p-cymen-7-ol
27	10.8882	2.80	2-methoxy-4-vinylphenol (or 4-vinylguaiacol)
28	11.2193	0.98	DL-arabinose
29	11.7451	1.09	DL-proline, 5-oxo-, methyl ester
30	12.0470	0.74	vanillin
31	12.6702	1.96	2-methoxy-4-(1-propenyl)-phenol (Z)- (or *cis*-isoeugenol)
32	13.1473	1.05	1-[4-(methylthio)phenyl]-ethanone
33	13.4492	0.86	butylated hydroxytoluene
34	13.6877	0.57	benzeneacetic acid, 4-hydroxy-3-methoxy-, methyl ester
35	13.9458	0.82	1,4-diacetyl-3-acetoxymethyl-2,5-methylene-l-rhamnitol
36	14.3693	2.44	α-methyl-l-sorboside
37	15.0461	5.78	guanosine
38	15.5865	8.31	1,4-diacetyl-3-acetoxymethyl-2,5-methylene-l-rhamnitol
39	16.0734	1.65	4-((1E)-3-hydroxy-1-propenyl)-2-methoxyphenol (or coniferyl alcohol)
40	17.4561	0.43	5-amino-1-(4-amino-furazan-3-yl)-1H-[1,2,3]triazole-4-carbonitrile
41	17.9088	1.18	hexadecanoic acid, methyl ester
42	18.2594	1.17	n-hexadecanoic acid
43	19.1552	0.76	5-(1,1-dimethylethyl)[1,1′-biphenyl]-2-ol (or 4-tert-butyl-2-phenylphenol)
45	19.4278	0.69	cyclopentadecane
46	19.6421	0.15	1-hydroxy-9,10-anthracenedione (or *α*-hydroxyanthraquinone)
47	19.8709	15.54	9,10-anthracenedione, 2-methyl- (or *β*-methylanthraquinone)
48	20.7278	1.43	1-hydroxy-4-methylanthraquinone
49	21.1659	1.75	1,2-dihydroxyanthraquinone (or alizarin)
50	21.8038	1.40	azacyclotridecan-2-one, 1-(3-aminopropyl)-
51	23.0550	0.81	glycerol 1-palmitate
52	23.3812	0.73	bis(2-ethylhexyl) phthalate
53	23.8973	0.24	1,8-dihydroxy-3-methyl-9,10-anthracenedione (or 1,8-dihydroxy-3-methyl anthraquinone)
54	24.2673	8.57	4-methoxy-4′,5′-methylenedioxybiphenyl-2-carboxylic acid
55	24.4474	0.42	9-octadecenoic acid (Z)-, 2-hydroxy-1-(hydroxymethyl)ethyl ester
56	25.4260	0.35	squalene
57	29.2333	0.62	octasiloxane, 1,1,3,3,5,5,7,7,9,9,11,11,13,13,15,15-hexadecamethyl-
58	29.9051	0.40	*γ*-sitosterol

**Table 4:** EC_50_ and EC_90_ effective concentrations. Values are expressed in µg·mL^−1^, and are followed by the standard errors of fit.

Pathogen	EC	COS	Stevioside	*R. tinctorum*	COS— *R. tinctorum*	Stevioside —*R. tinctorum*	4-*tert*…	1,2,4-trihydro…	Guanosine
*D. seriata*	EC_50_	744.4 ± 43.9	288.1 ± 15.3	78.0 ± 0.8	63.1 ± 0.3	73.6 ± 0.3	53.0 ± 2.1	45.4 ± 3.4	130.4 ± 12.8
	EC_90_	1179.9 ± 58.2	840.5 ± 62.3	87.8 ± 1.9	73.4 ± 0.9	82.4 ±0.7	73.2 ± 2.3	171.4 ± 18.7	249.9 ± 28.5
*D. viticola*	EC_50_	554.3 ± 27.4	306.9 ± 26.6	66.2 ± 2.9	22.1 ± 1.4	80.0 ± 0.7	25.7 ± 3.6	37.2 *	182.7 ± 7.7
	EC_90_	1138.7 ± 75.0	917.0 ± 74.3	90.2 ± 8.7	55.5 ± 4.6	90.7 ± 1.5	71.2 ± 9.0	74.9 *	308.1 ± 23.7
*N. parvum*	EC_50_	680.2 ± 43.1	194.8 ± 13.4	92.3 ± 0.5	38.2 ± 1.4	75.1 ± 0.8	62.2 ± 0.7	72.0 ± 14.8	95.1 ± 22.6
	EC_90_	1326.6 ± 83.2	723.8 ± 56.7	184.0 ± 1.1	66.3 ± 4.2	89.2 ± 1.9	70.6 ± 2.2	338.4 ± 37.9	317.8 ± 33.9

* Could not be reliably calculated (lack of points).

**Table 5:** Synergy factors, estimated according to Wadley’s method.

Pathogen	EC	Synergy Factor	
		COS-*R. tinctorum*	Stevioside—*R. tinctorum*
*D. seriata*	EC_50_	2.24	1.67
	EC_90_	2.23	1.93
*D. viticola*	EC_50_	5.35	1.36
	EC_90_	3.01	1.81
*N. parvum*	EC_50_	4.26	1.67
	EC_90_	4.87	3.29

**Table 6:** Kruskal–Wallis test and multiple pairwise comparisons using the Conover–Iman procedure for the lengths of the vascular necroses scored for *D. seriata* in the ex situ autoclaved grapevine canes assay.

Sample	Frequency	Sum of Ranks	Mean of Ranks	Groups		
COS-*R. tinctorum* negative control	24	300.000	12.500	A		
COS-*R. tinctorum-D. seriata*	120	10,193.000	84.942		B	
Positive control	24	3703.000	154.292			C

Treatments/controls labelled with the different letters are significantly different at *p* < 0.05.

**Table 7:** Kruskal-Wallis test and multiple pairwise comparisons using the Conover–Iman procedure for the lengths of the vascular necroses for the three phytopathogen in greenhouse in vivo assays.

Pathogen	Sample	Frequency	Sum of Ranks	Mean of Ranks	Groups		
*D. seriata*	COS-*R. tinctorum* negative control	32	725.500	22.672	A		
	COS-*R. tinctorum-D. seriata*	72	6124.000	85.056		B	
	Positive control	56	6030.500	107.688			C
*D. viticola*	COS-*R. tinctorum* negative control	32	1295.000	40.469	A		
	COS-*R. tinctorum-D. viticola*	72	4885.000	67.847		B	
	Positive control	64	8016.000	125.250			C
*N. parvum*	COS-*R. tinctorum* negative control	32	572.000	17.875	A		
	COS-*R. tinctorum-N. parvum*	48	3695.000	76.979		B	
	Positive control	64	6173.000	96.453			C

Treatments/controls labelled with the different letters are significantly different at *p* < 0.05.

## Data Availability

The data presented in this study are available on request from the corresponding author. The data are not publicly available due to their relevance to an ongoing Ph.D. thesis.

## Supplementary Materials

The following are available online at https://www.mdpi.com/article/10.3390/plants10081527/s1, Table S1. Repetitions for each of the plant/treatment combinations in the greenhouse bioassay. Each grafted plant was inoculated at two sites below grafting point; Table S2. Examples of application of *R. tinctorum* extracts against microorganisms reported in the literature; Figure S1. GC–MS spectrum of *R. tinctorum* root hydromethanolic extract; Figures S2–S4. Mycelial growth inhibition of *D. seriata*/*D. viticola*/*N. parvum* upon treatment with: chitosan oligomers, stevioside, *R. tinctorum* hydromethanolic extract, stevioside–*R. tinctorum* conjugate complex, and COS-*R. tinctorum* conjugate complex at different concentrations; Figure S5. Colony growth measures of *D. seriata*, *D. viticola* and *N. parvum* strains when cultured in PDA plates containing the main phytochemicals found in *R. tinctorum* hydromethanolic extracts at concentrations in the 62.5–1500 and 15.62–250 μg·mL^−1^ range for the least and the most active products, respectively; Figures S6–S8. Mycelial growth inhibition of *D. seriata*/*D. viticola*/*N. parvum* upon treatment with the main phytochemicals found in *R. tinctorum* hydromethanolic extracts: purpurin, guanosine, and 4-*tert*-butyl-2-phenylphenol, at different concentrations.
