# Supplementary material for: Activity of Anthracenediones and Flavoring Phenols in Hydromethanolic Extracts of Rubia tinctorum against Grapevine Phytopathogenic Fungi
Source: Plants (Basel). 2021 Jul 26;10(8):1527. doi: 10.3390/plants10081527 (PMC8399478; doi:10.3390/plants10081527)
Supplement: Supplementary file 1 [file plants-10-01527-s001.zip › plants-1311978-supplementary.pdf]

Activity of anthracenediones and flavoring phenols in hydromethanolic extracts of *Rubia tinctorum* against grapevine phytopathogenic fungi

Natalia Langa-Lomba, Eva Sánchez-Hernández, Laura Buzón-Durán, Vicente González-García, José Casanova-Gascón, Jesús Martín-Gil, and Pablo Martín-Ramos

SUPPORTING INFORMATION

Table S1. Repetitions for each of the plant/treatment combinations in the greenhouse bioassay. Each grafted plant was inoculated at two sites below grafting point.

Plant	Treatment	Pathogen	Number of replicates
'Tempranillo' (CL. 32 clone) on 775P rootstock	COS-*R. tinctorum*	*D. seriata*	5
		D. viticola	5
		N. parvum	5
		None (negative control)	3
	None (positive control)	*D. seriata*	4
		D. viticola	4
		N. parvum	4
'Garnacha' (VCR3 clone) on 110R rootstock	COS-*R. tinctorum*	*D. seriata*	5
		D. viticola	5
		N. parvum	5
		None (negative control)	3
	None (positive control)	*D. seriata*	4
		D. viticola	4
		N. parvum	4

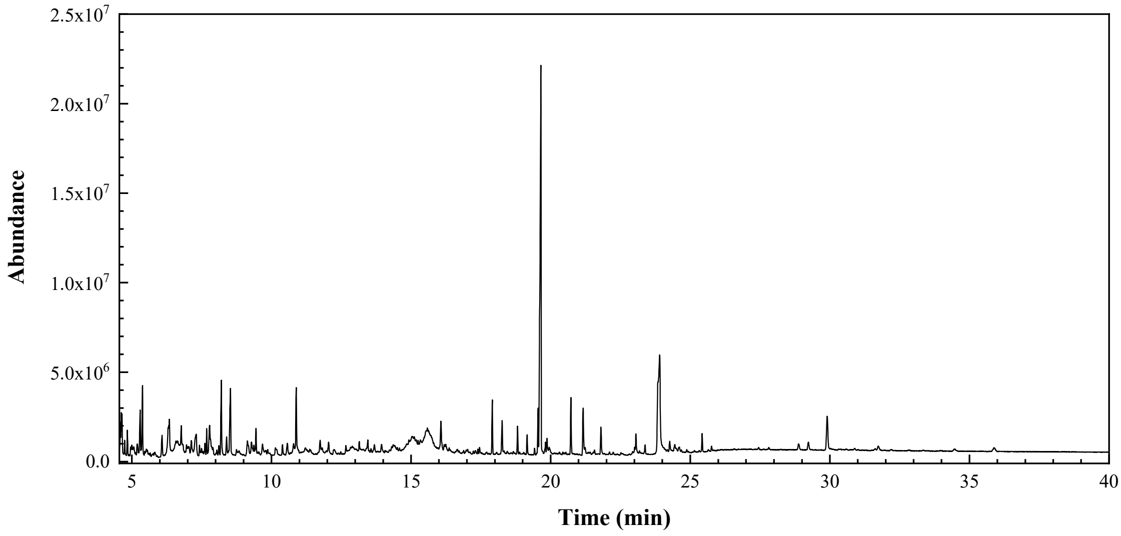

Figure S1. GC-MS spectrum of *R. tinctorum* root hydromethanolic extract

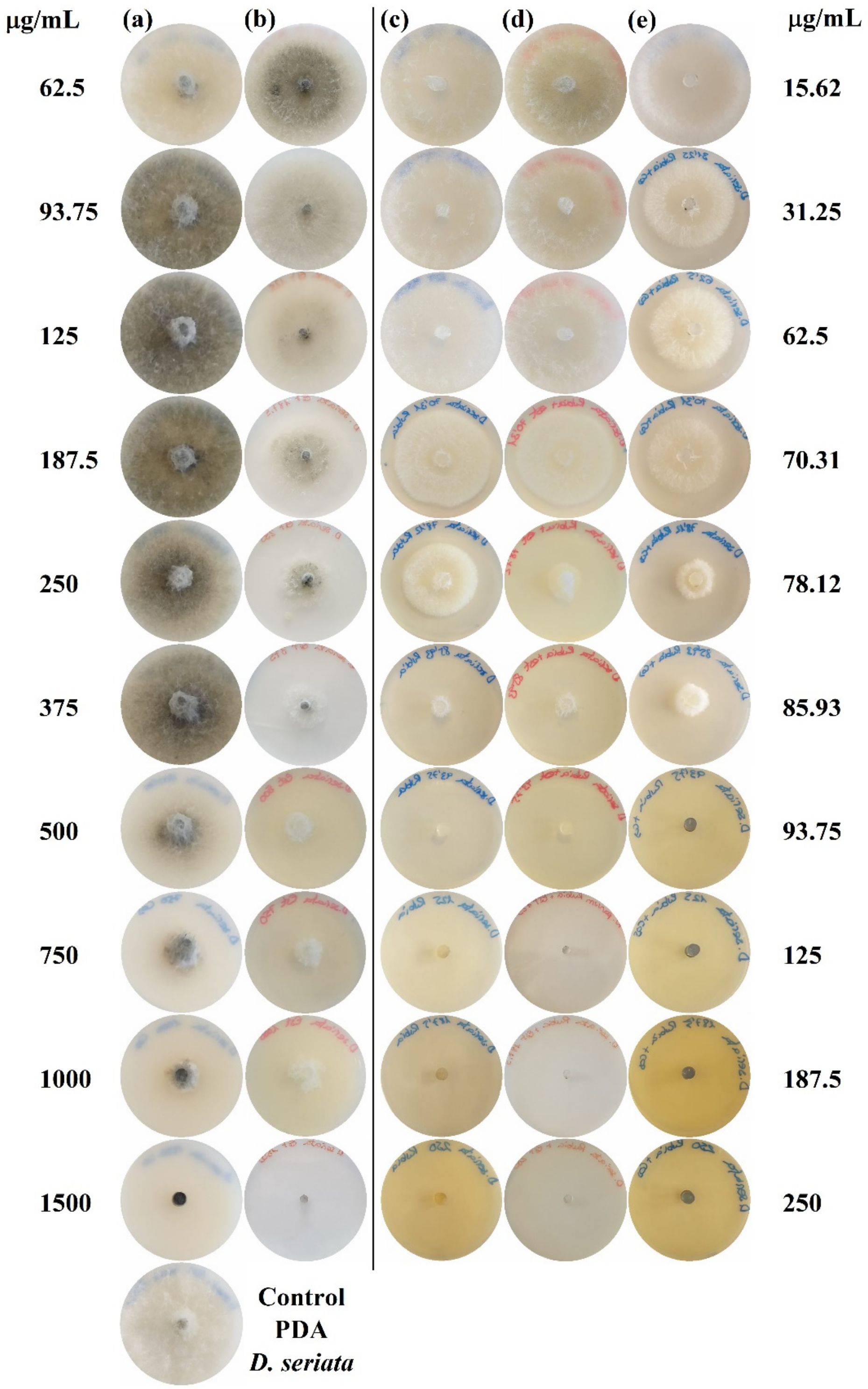

Figure S2. Mycelial growth inhibition of *D. seriata* upon treatment with: **(a)** chitosan oligomers, COS; **(b)** stevioside; **(c)** *R. tinctorum* hydromethanolic extract; **(d)** stevioside–*R. tinctorum* conjugate complex; **(e)** COS–*R. tinctorum* conjugate complex at different concentrations. Only one replicate is shown.

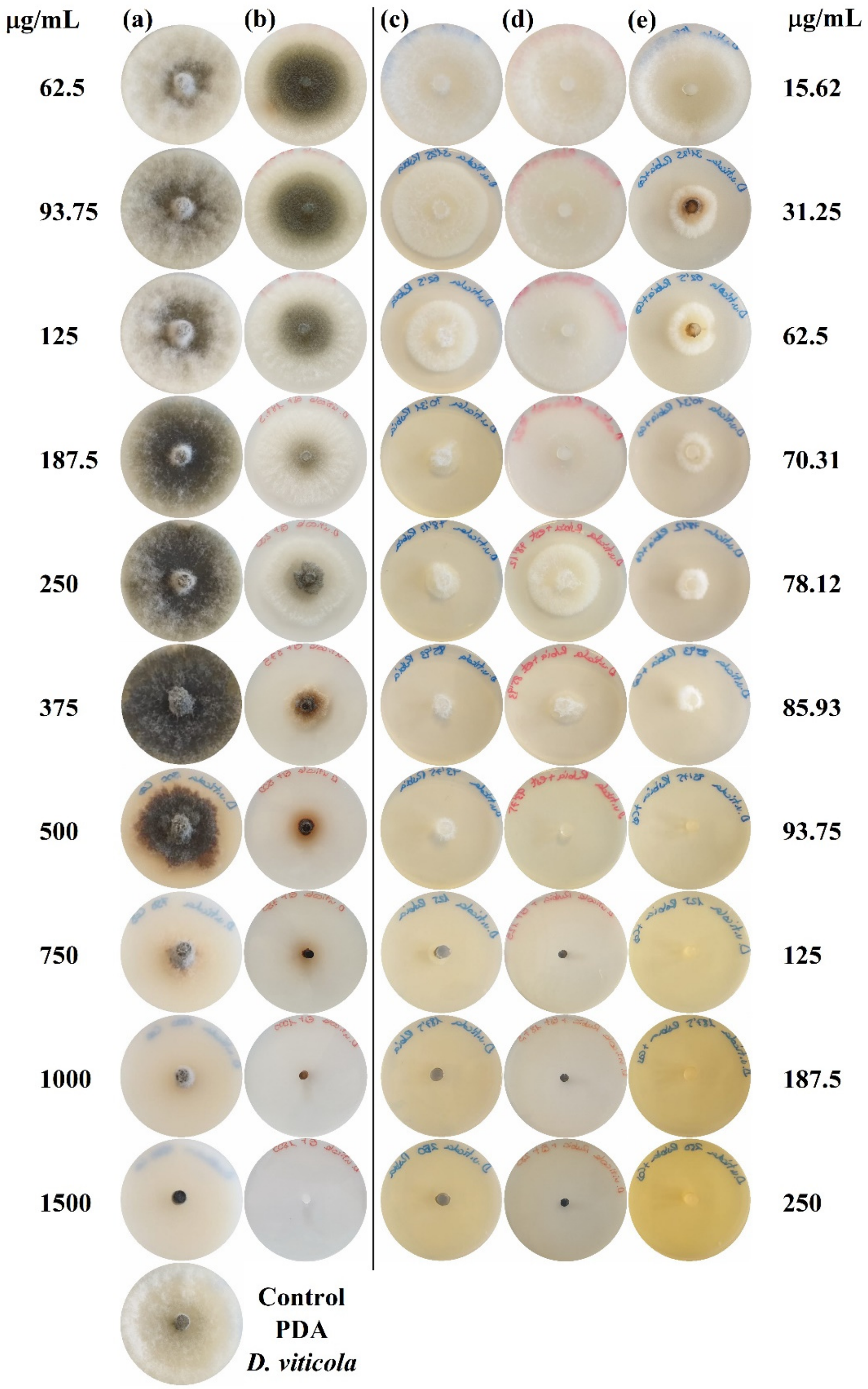

Figure S3. Mycelial growth inhibition of *D. viticola* upon treatment with: **(a)** chitosan oligomers, COS; **(b)** stevioside; **(c)** *R. tinctorum* hydromethanolic extract; **(d)** stevioside–*R. tinctorum* conjugate complex; **(e)** COS–*R. tinctorum* conjugate complex at different concentrations. Only one replicate is shown.

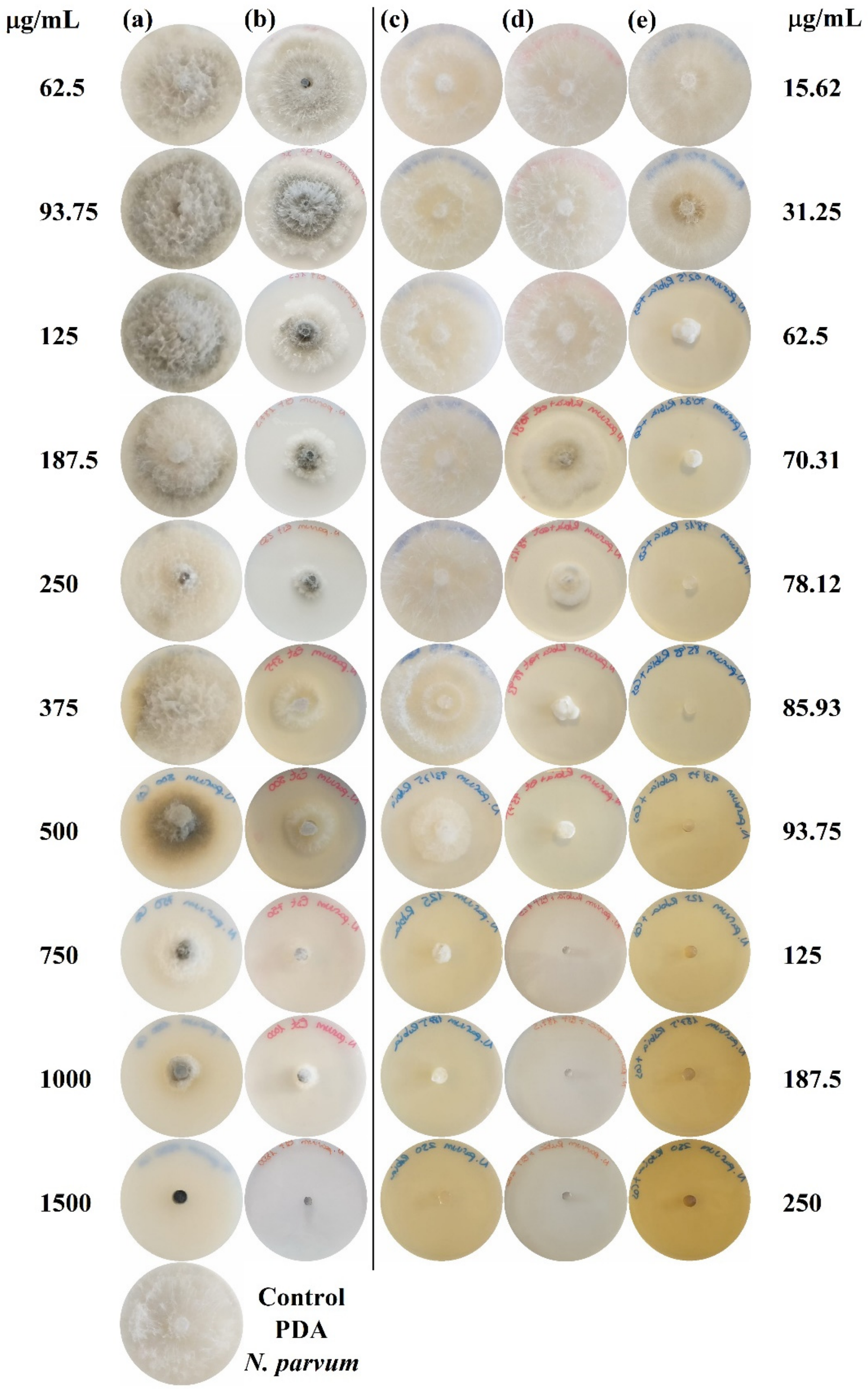

Figure S4. Mycelial growth inhibition of *N. parvum* upon treatment with: **(a)** chitosan oligomers, COS; **(b)** stevioside; **(c)** *R. tinctorum* hydromethanolic extract; **(d)** stevioside–*R. tinctorum* conjugate complex; **(e)** COS–*R. tinctorum* conjugate complex, at different concentrations. Only one replicate is shown.

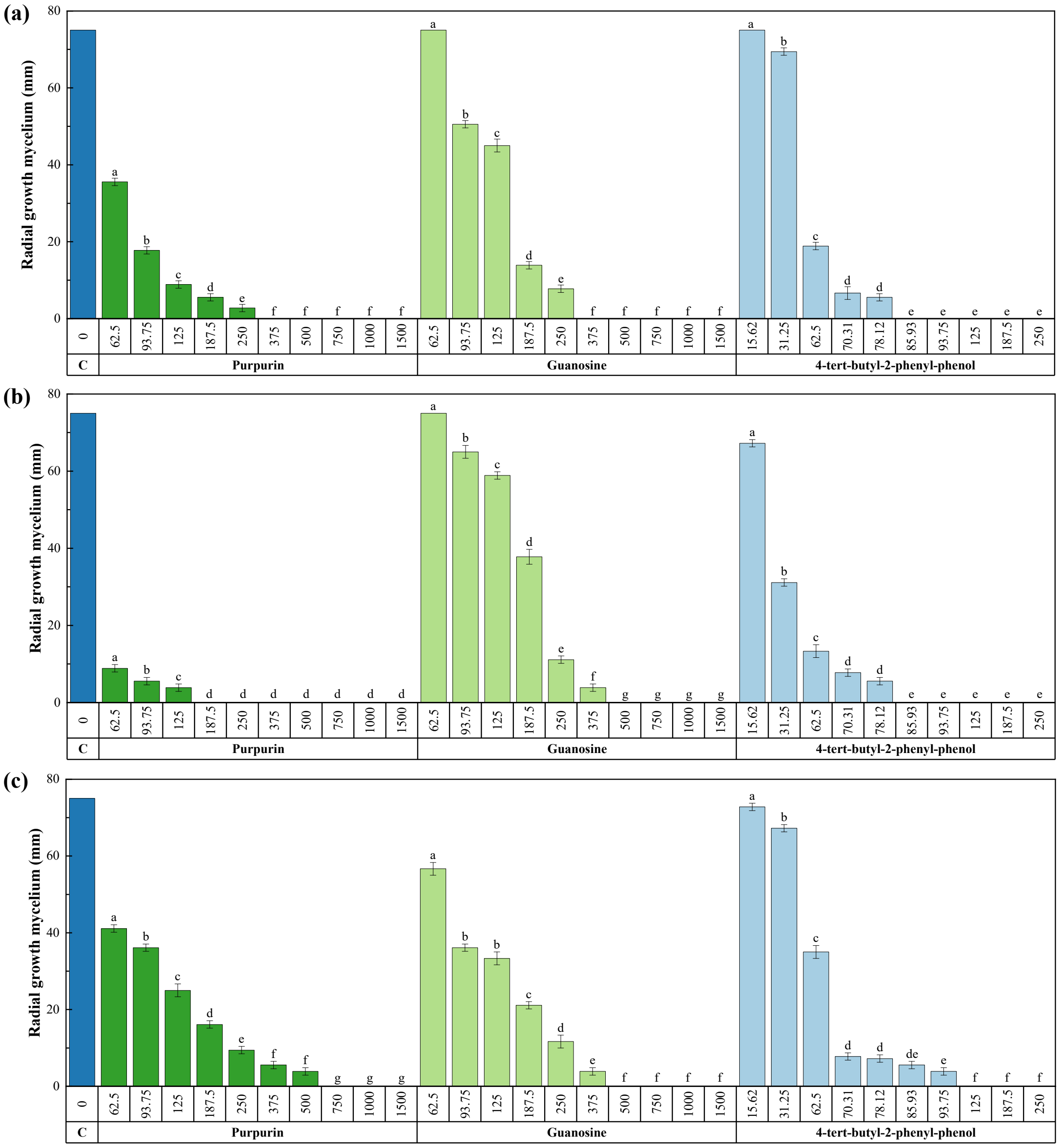

Figure S5. Colony growth measures of (**a**) *D. seriata*, (**b**) *D. viticola* and (**c**) *N. parvum* strains when cultured in PDA plates containing the main phytochemicals found in *R. tinctorum* hydromethanolic extracts (viz. purpurin, guanosine and 4-*tert*-butyl-2-phenyl-phenol) at concentrations in the 62.5–1500 and 15.62–250 μg·mL^{-1} range for the least and the most active products, respectively. The same letters above concentrations indicate that they are not significantly different at $p < 0.05$. Error bars represent standard deviations.

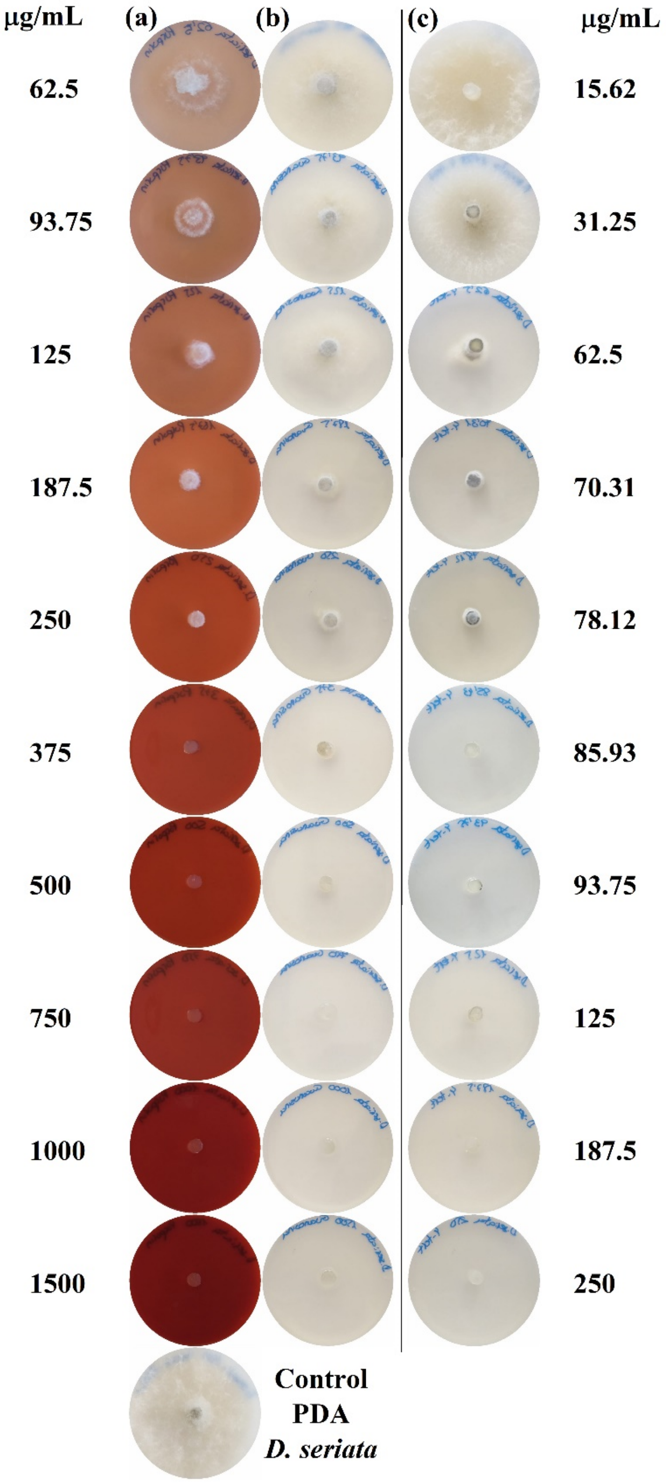

Figure S6. Mycelial growth inhibition of *D. seriata* upon treatment with the main phytochemicals found in *R. tinctorum* hydromethanolic extracts: **(a)** purpurin, **(b)** guanosine, and **(c)** 4-tert-butyl-2-phenyl-phenol, at different concentrations. Only one replicate is shown.

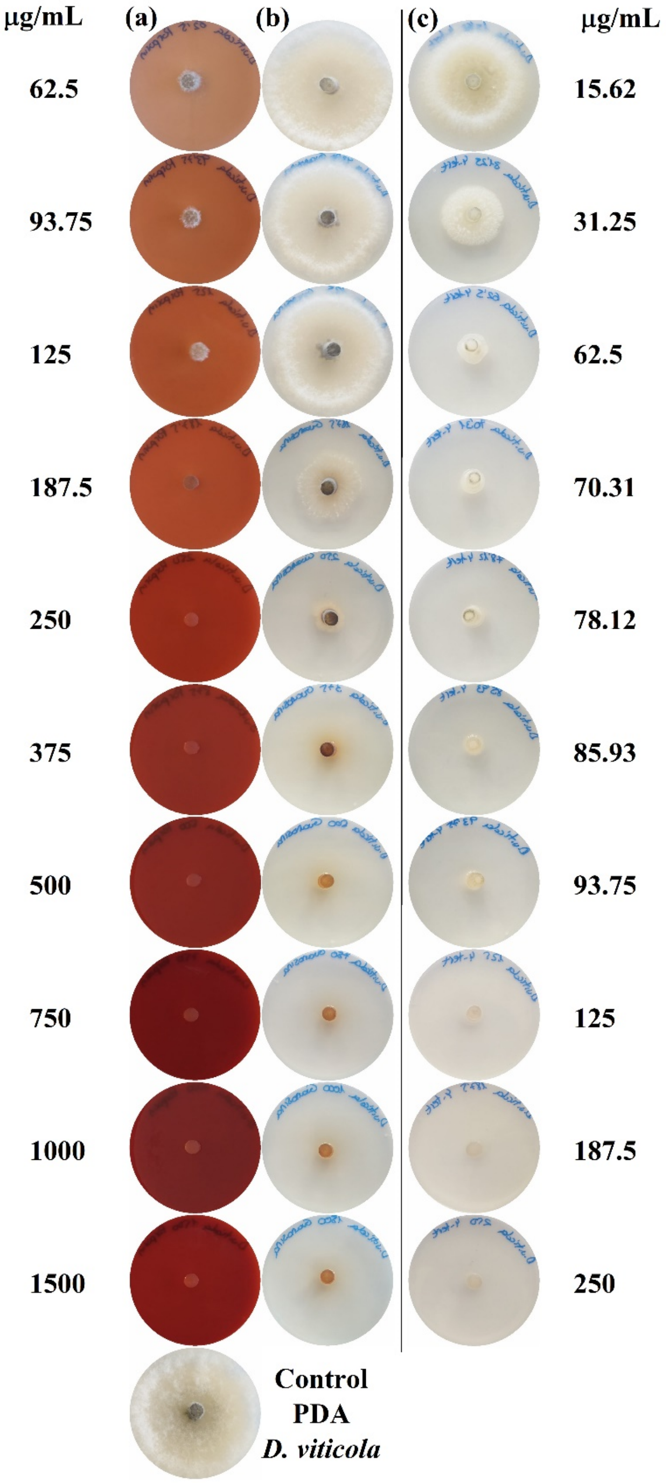

Figure S7. Mycelial growth inhibition of *D. viticola* upon treatment with the main phytochemicals found in *R. tinctorum* hydromethanolic extracts: **(a)** purpurin, **(b)** guanosine, and **(c)** 4-tert-butyl-2-phenyl-phenol, at different concentrations. Only one replicate is shown.

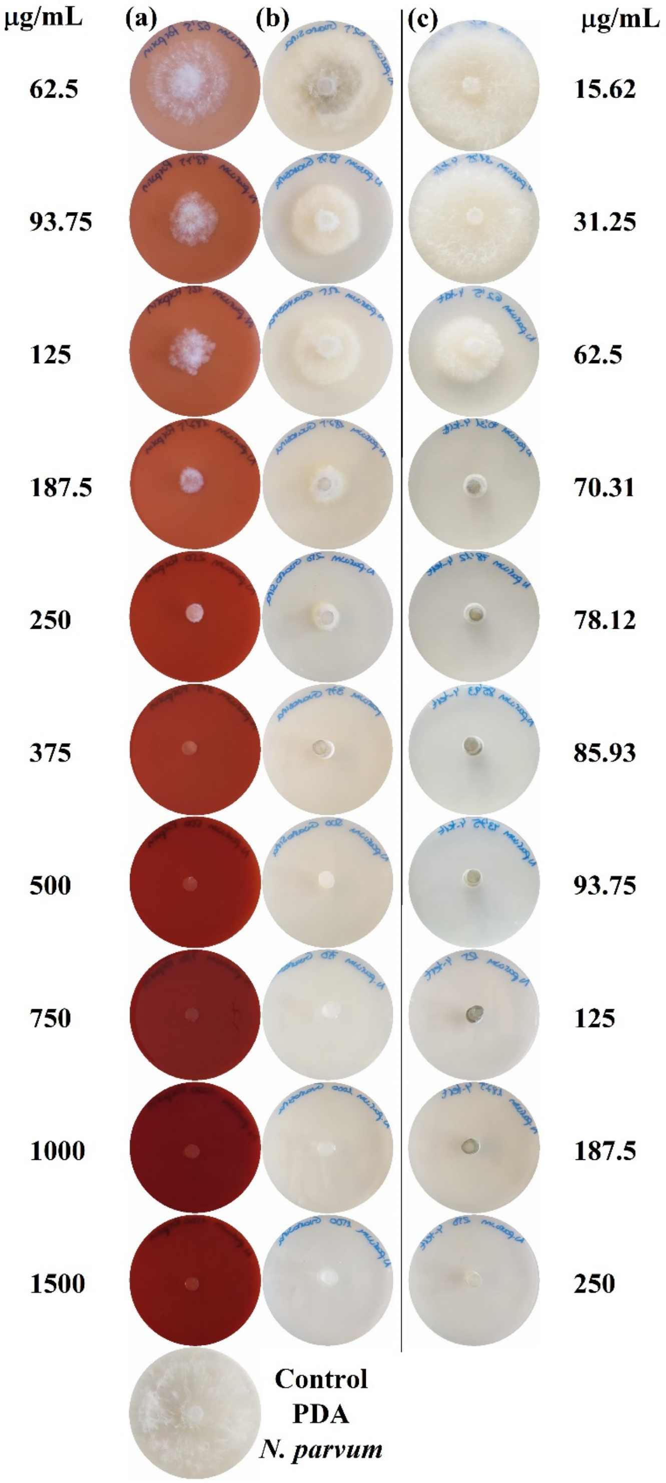

Figure S8. Mycelial growth inhibition of *N. parvum* upon treatment with the main phytochemicals found in *R. tinctorum* hydromethanolic extracts: **(a)** purpurin, **(b)** guanosine, and **(c)** 4-tert-butyl-2-phenyl-phenol, at different concentrations. Only one replicate is shown.

Table S2. Examples of application of *R. tinctorum* extracts against microorganisms reported in the literature.

Pathogen	Assayed product	Effectiveness	Ref.
Antibacterial activity			
Shigella dysenteriae	Aqueous extract	MIC = 25-100 mg·mL^{-1}	[1]
Aeromonas hydrophila	Methanol extract	MIC = 5-50 mg·mL^{-1}	
Escherichia coli			
Klebsiella spp.			
Serratia marcescens			
Staphylococcus aureus			
	Aqueous solution of commercial natural dye powder (10 g/300 mL)	Inhibition zone (mm)	[2]
Bacillus cereus RSKK 863		-	
Bacillus megaterium RSKK 5117		8.5	
Shigella sonnei RSKK 877		-	
Staphylococcus aureus ATCC 25923		9.7	
Bacillus subtilis RSKK 244		10.2	
Pseudomonas aeruginosa ATCC 29212		-	
Salmonella sp. 21.3		-	
P. aeruginosa ATCC 27853		-	
Streptococcus epidermidis		-	
	Methanolic extract, without / with acid hydrolysis at 1 mg·mL^{-1}	Inhibition diameter (mm)	[3]
Staphylococcus aureus ATCC 25923		17.0±0.7 / 19.4±0.5	
Listeria monocytogenes ATCC 11120		19.3±0.9 / 21.1±0.7	
Pseudomonas aeruginosa ATCC 9027		14.7±0.3 / 16.6±0.4	
Salmonella enteritidis ATCC 14028		12.2±0.7 / 15.5±0.0	
Escherichia coli ATCC 25922		10.1±0.3 / 11.2±0.6	
Aeromonas hydrophila ATCC 1943		13.4±0.8 / 14.3±0.1	
Klebsiella pneumoniae ATCC 13833		13.0±0.5 / 15.5±0.0	
Staphylococcus aureus	Methanolic extract	MIC = 0.1562 mg·mL^{-1}	[4]
Bacillus subtilis		MIC = 0.1562 mg·mL^{-1}	
Escherichia coli		MIC = 0.3125 mg·mL^{-1}	
Pseudomonas aeruginosa		MIC = 0.3125 mg·mL^{-1}	
Escherichia coli ATCC 11230	Ethanol, methanol, ethyl acetate and water extracts (5 mg/mL)	+	[5]
Enterobacter aerogenes ATCC 13048		+	
Proteus vulgaris ATCC 8427		-	
Serratia marcescens NRRL 3284		-	
Bacillus cereus ATCC 7064		+	
Bacillus subtilis ATCC 6633		+	
Micrococcus luteus LA 2971		+	
Staphylococcus aureus ATCC 6538P		+	
Klebsiella pneumoniae UC57		-	
Pseudomonas aeruginosa ATCC 27853		+	
Bacillus subtilis	aqueous / methanolic extracts (50 g/100 mL)	+	[6]
Bacillus cereus		+	
Bacillus mycoides		+	
Escherichia coli ATCC 25922	Aqueous / ethanolic extracts	MIC = 500 / 1000 μg·mL^{-1}	[7]
Staphylococcus aureus ATCC 6538	Silk dyed with madder	+	[8]
Escherichia coli ATCC 25922		-	
Staphylococcus aureus	PET fabric dyed with madder	86% inhibition	[9]
Escherichia coli			
Antifungal activity			
Candida albicans DSMZ 1386	Silk dyed with madder	None	[10]
	Methanolic extract, with and without acid hydrolysis (1 mg/mL)	Inhibition diameter	[3]
Aspergillus niger		19.6±0.8 / 21.4±0.8 mm	
Aspergillus ochraceus		17.9±0.1 / 19.6±0.4 mm	
Candida albicans ATCC 10231	Ethanol, methanol, ethyl acetate and water extracts (5 mg/mL for yeasts, 10-100 μg/mL for filamentous fungi)	+	[5]
Saccharomyces cerevisiae ATCC 9763		+	
Geotrichum penicillatum		-	
Aspergillus flavus		Full inhibition at 100 μg·mL^{-1}	
Fusarium oxysporum		Full inhibition at 100 μg·mL^{-1}	
Candida spp.	Purpurin	MIC = 1.28–5.12 μg·mL^{-1}	[11]

	R. tinctorum roots methanolic extract / alizarin (concentration not reported)	% inhibition	[12]
Trichoderma viride		43 / 50	
Doratomyces stemonitis		41 / 20	
Aspergillus niger		22 / 20	
Penicillium verrucosum		35 / 22	
Alternaria alternata		18 / 18	
Aueobasidium pullulans		18 / 14	
Mucor mucedo		22 / 31	
Penicillium expansum	aqueous / methanolic extracts	+ / +	[6]
Aspergillus niger		+ / +	
Alternaria alternata		+ / +	
Geotrichum candidum		+ / +	
Fusarium solani		+ / +	
Postia placenta	Wood treated with aqueous extract (1:20 mass plant material to liquid)	+	[13]
Trametes versicolor		+	
Actinomycetes			
Streptomyces murinus ISP 5091	Ethanol, methanol, ethyl acetate and water extracts (10-100 µg/mL)	Full inhibition at 100 $\mu g \cdot mL^{-1}$	[5]

References

1. Aboud, A.S. HPLC analysis of *Rubia tinctorum* and its effect of methanol and aqueous extract on bacteria isolated from burns infection. *Al-Nahrain Journal of Science* **2010**, *13*, 166-175.
2. Calis, A.; Celik, G.Y.; Katircioglu, H. Antimicrobial effect of natural dyes on some pathogenic bacteria. *African Journal of Biotechnology* **2009**, *8*, 291-293.
3. Essaidi, I.; Snoussi, A.; Koubaier, H.B.H.; Casabianca, H.; Bouzouita, N. Effect of acid hydrolysis on alizarin content, antioxidant and antimicrobial activities of *Rubia tinctorum* extracts. *Pigment & Resin Technology* **2017**, *46*, 379-384, doi:10.1108/prt-11-2015-0116.
4. Ghafari, R.; Mouslemanie, N.; Nayal, R. Antibacterial activity of *Rubia tinctorum* Linn. root extracts. *International Journal of Pharmaceutical Sciences and Research* **2018**, *9*, 3914-3918.
5. Kalyoncu, F.; Cetin, B.; Saglam, H. Antimicrobial activity of common madder (*Rubia tinctorum* L.). *Phytother Res* **2006**, *20*, 490-492, doi:10.1002/ptr.1884.
6. Mehrabian, S.; Majd, A.; Majd, I. Antimicrobial effects of three plants (*Rubia tinctorum, Carthamus tinctorius* and *Juglans regia*) on some airborne microorganisms. *Aerobiologia* **2000**, *16*, 455-458, doi:10.1023/a:1026571914665.
7. Rovcanin, B.R.; Cebovic, T.; Stesevic, D.; Kekic, D.; Ristic, M. Antibacterial effect of *Herniaria hirsuta, Prunus avium, Rubia tinctorum* and *Sempervivum tectorum* plant extracts on multiple antibiotic resistant *Escherichia coli*. *Bioscience Journal* **2015**, *31*, 1852-1861.
8. Guzel, E.T.; Karadag, R.; Alkan, R. Durability, antimicrobial activity and HPLC analysis of dyed silk fabrics using madder and gall oak. *Journal of Natural Fibers* **2020**, *17*, 1654-1667, doi:10.1080/15440478.2019.1588827.
9. Agnhage, T.; Zhou, Y.; Guan, J.; Chen, G.; Perwuelz, A.; Behary, N.; Nierstrasz, V. Bioactive and multifunctional textile using plant-based madder dye: Characterization of UV protection ability and antibacterial activity. *Fibers and Polymers* **2017**, *18*, 2170-2175, doi:10.1007/s12221-017-7115-x.
10. Alkan, R.; Torgan, E.; Karadag, R. The investigation of antifungal activity and durability of natural silk fabrics dyed with madder and gallnut. *Journal of Natural Fibers* **2017**, *14*, 769-780, doi:10.1080/15440478.2017.1279101.
11. Kang, K.; Fong, W.-P.; Tsang, P.W.-K. Novel antifungal activity of purpurin against *Candida* species in vitro. *Medical Mycology* **2010**, *48*, 904-911, doi:10.3109/13693781003739351.
12. Manojlovic, N.T.; Solujic, S.; Sukdolak, S.; Milosev, M. Antifungal activity of *Rubia tinctorum*, *Rhamnus frangula* and *Caloplaca cerina*. *Fitoterapia* **2005**, *76*, 244-246, doi:10.1016/j.fitote.2004.12.002.
13. Ozen, E.; Yeniocak, M.; Goktas, O.; Alma, M.H.; Yilmaz, F. Antimicrobial and antifungal properties of madder root (*Rubia tinctorum*) colorant used as an environmentally-friendly wood preservative. *Bioresources* **2014**, *9*, 1998-2009.
